# Interleukin-34 promotes tumorigenic signals for colon cancer cells

**DOI:** 10.1038/s41420-021-00636-4

**Published:** 2021-09-17

**Authors:** Eleonora Franzè, Irene Marafini, Edoardo Troncone, Silvia Salvatori, Giovanni Monteleone

**Affiliations:** Department of Systems Medicine, University of Rome “TOR VERGATA”, Rome, Italy

**Keywords:** Preclinical research, Inflammatory bowel disease

## Abstract

Colorectal carcinoma (CRC) is one of the most common forms of malignancy in the Western world. Accumulating evidence indicates that colon carcinogenesis is tightly controlled by tumour-associated immune cells and stromal cells, which can either stimulate or suppress CRC cell growth and survival, mainly via the production of cytokines. Interleukin-34 (IL-34), a cytokine known to regulate mainly monocyte/macrophage survival and function, is highly produced within the CRC microenvironment by several cell types, including cancer cells, tumour-associated macrophages (TAMs) and cancer-associated fibroblasts (CAFs), and regulates the pro-tumoural functions of such cells. In this article, we summarize the available data supporting the multiple effects of IL-34 in human CRC.

## Facts

- IL-34 is a positive regulator of colorectal cancer (CRC) cell growth.
- IL-34 acts on CRC cells as well as on other immune cells and non-immune cells (i.e. tumour-associated macrophages and cancer-associated fibroblasts) in the CRC microenvironment.
- Association between high tissue expression of IL-34 and unfavourable prognosis and poor survival in CRC patients has been documented.

## Open questions

- Can IL-34 blockers enhance the properties of other anti-tumoural drugs?
- Can circulating IL-34 serve as a prognostic biomarker in colorectal cancer?

## Introduction

Colorectal cancer (CRC) is the third most commonly diagnosed form of cancer globally, accounting for approximately 10% of all annually diagnosed cancers worldwide. Incidence rates vary geographically with highest frequencies in developed countries [1, 2]. Despite significant advances in prevention and diagnosis, CRC is still one of the most deadly cancers worldwide, and this is because in about one fourth of the patients CRC diagnosis is made when cancer has metastasized and surgery, which remains the primary course of treatment in cases of early diagnosis, is no longer effective [2].

The CRC actually comprises a heterogeneous group of neoplasias, which are associated with different risk factors. More than two thirds of CRC arise sporadically and environmental and demographic factors (e.g. smoking habit, red meat consumption and obesity, age, positive family history of CRC) are supposed to play a key role in the pathogenesis of this form of CRC [3]. A small group of patients (5–7%) have a well-defined hereditary CRC syndrome, such as hereditary nonpolyposis colorectal cancer and familial adenomatous polyposis [4], while in 2–3% of cases CRC arises in patients with long-standing inflammatory bowel diseases (IBD) [5, 6].

CRC arises when colonic epithelial cells acquire a series of genetic or epigenetic mutations that increase cell growth and survival. Support to the abnormal behaviour of cancer cells is given by immune cells and stromal cells, which produce several pro-tumorigenic factors. On the other hand, cancer cells secrete several chemoattractants for immune cells [7]. Moreover, CRC cells synthesize a large array of cytokines, which enhance the pro-tumoural functions of immune cells and stromal cells, thus contributing to generate a microenvironment that favours disease progression [7]. One such a molecule is interleukin-34 (IL-34), a cytokine that was initially known as a factor regulating survival, proliferation and differentiation of monocytes, macrophages, and osteoclasts [8].

We here review the data about the expression and role of IL-34 in CRC.

## Interleukin-34 expression and signalling

In 2008, Lin and colleagues showed that macrophage colony-stimulating factor receptor (M-CSF-1-R) could bind, in addition to the well-known ligand macrophage colony-stimulating factor (M-CSF-1), IL-34 [8]. The mature, full-length human IL-34 protein comprises 242 amino acids with a molecular mass of 39 KDa [8]. Non-covalently linked IL-34 homodimer recruits two M-CSF1-R. Despite IL-34 and M-CSF-1 share the same receptor, they bind different anchorage points of M-CSFR-1 and activate distinctive signalling pathways thus mediating unique biological functions [9–13]. The distinct biological functions of IL-34 and M-CSF-1 are dependent on the different hydrophobic/hydrophilic interactions of each ligand with M-CSF1-R. In particular, the M-CSF-1:M-CSF1-R complex depends on hydrophilic interactions, while the IL-34:M-CSF1-R interface contains a large number of hydrophobic regions, which stabilize the cytokine-receptor binding and favour a prolonged and strong transmembrane signalling [8–11].

Binding of IL-34 to M-CSF1-R activates different signalling pathways [(i.e. nuclear factor kappa-light-chain-enhancer of activated B cells (NF-κB), phosphoinositide 3-kinase (PI3K)/AKT, p38 mitogen-activated protein kinase (MAPK), extracellular signal-regulated protein kinases 1 and 2 (ERK1/2), c-Jun N-terminal kinase (JNK), Janus kinase (JAK), signal transducer and activator of transcription (STAT)3], mostly depending on the cell type analysed [12–16]. In human primary monocytes, IL-34-induced signals can also activate caspase-3/8 and promote autophagy through an AMP-activated protein kinase-UNC-51-like Kinase 1-dependent mechanism [17].

**Fig. 1:**
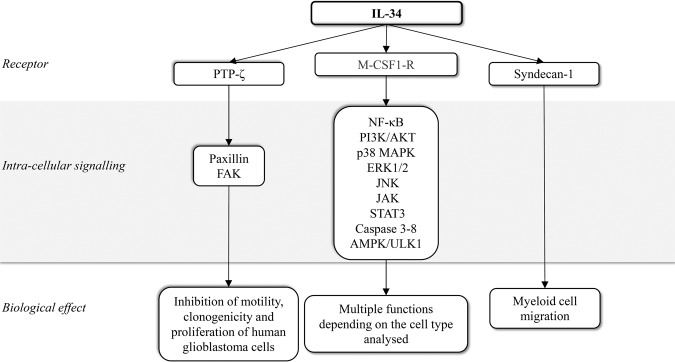
Schematic view of interleukin (IL)-34 receptors and IL-34-induced signalling pathways. M-CSF-1-R macrophage colony-stimulating factor receptor, NF-κB nuclear factor kappa-light-chain-enhancer of activated B cells, PI3K phosphoinositide 3-kinase, MAPK mitogen-activated protein kinase, ERK1/2 extracellular signal-regulated protein kinases 1 and 2, JNK c-Jun N-terminal kinase, JAK Janus kinase, STAT3 signal transducer and activator of transcription 3, ULK1 UNC-51-like Kinase 1, PTP-ζ protein-tyrosine phosphatase zeta, FAK focal adhesion kinase, TAMs tumour-associated macrophages.

A second receptor of IL-34 is receptor-type protein-tyrosine phosphatase zeta (PTP-ζ). PTP-ζ is a cell surface chondroitin sulfate proteoglycan primarily expressed on neuronal progenitors and glial cells and to a lesser extent on B cells and kidney tubular cells [18]. After interaction with IL-34, PTP-ζ induces a series of intracellular events that inhibit motility, clonogenicity, and proliferation of glioblastoma cells via tyrosine phosphorylation of paxillin and focal adhesion kinase [18]. The third and last functional IL-34 receptor identified is Syndecan-1 (also known as CD138) that, once engaged by IL-34, stimulates myeloid cell migration [19] (Fig. 1).

IL-34 is produced by a wide range of cell types, including macrophages, endothelial cells, fibroblasts, adipocytes, neurons, cancer cells, and epithelial cells and is constitutively expressed in adult human tissues, such as heart, brain, testis, ovary, prostate, spleen, liver, thymus, small intestine, and colon [20–26]. IL-34 levels can change under pathological conditions [27]. Increased IL-34 RNA and protein expression was documented in various diseases, including autoimmune and inflammatory disorders, infections, metabolic diseases, neurological disorders, and fibrosis while a reduced expression of the cytokine was documented in Alzheimer’s disease, atopic dermatitis, hepatitis B viral infection, and periodontal diseases [23, 26, 28–38] (Table 1). Changes in IL-34 expression have also documented in various neoplastic diseases, where the cytokine is supposed to either limit or enhance the carcinogenetic processes [20, 39–51] (Table 2). IL-34 expression can be upregulated by several stimuli [23, 26, 34, 52]. Inflammatory cytokines, such as tumour necrosis factor (TNF)-α, IL-6 and IL-1β, activate NF-κB, JNK, and ERK1/2 signalling pathways and enhance IL-34 synthesis in a wide range of cell types including fibroblasts, epithelial cells, intestinal lamina propria mononuclear cells (LPMC), periodontal ligament cells, osteosarcoma cells, and adipocytes [15, 26, 34, 53]. Pathogen-associated molecular patterns, such as peptidoglycan, lipopolysaccharide and nucleic acids mimickers, poly I:C, and CpG, bind to Toll like receptors and induce IL-34 expression in macrophages, intestinal LPMC, and adipocytes. Iα,25(OH)2D3, a hormonally active form of vitamin D, increases IL-34 expression in neuroblastoma cells and normal gastric epithelial cells [26, 34, 52]. Infectious agents (i.e. hepatitis C virus) can increase IL-34 expression in infected cells [23]. On the contrary, transforming growth factor (TGF)-β1 and bone morphogenetic protein-2 can downregulate IL-34 expression in TNF-α-stimulated synovial fibroblasts and mesenchymal stem cells [54].

**Table 1:** Changes of IL-34 expression in human diseases.

Disease	IL-34 expression	References
Alzheimer’s disease	↓ mRNA	[29]
Ankylosing spondylitis	↑ protein	[31]
Atopic dermatitis	↓ mRNA	[30]
Periodontal diseases	↓ protein	[35]
Hepatitis B viral infection	↓ mRNA and protein	[32, 33]
Hepatitis C viral infection	↑ protein	[23]
Inflammatory bowel diseases	↑ mRNA and protein	[26]
Influenza A viral infection	↑ protein	[89]
Steatosis	↑ protein	[23]
Obesity	↑ protein	[34]
Psoriasis and psoriatic arthritis	↑ protein	[90]
Rheumatoid arthritis	↑ protein	[36, 37]
Sjogren’s syndrome	↑ mRNA and protein	[38]

Data are indicated as compared to controls (↑increased compared to controls; ↓decreased compared to controls).

**Table 2:** Role of IL-34 in different cancer types.

Cancer type	Function	References
Bone giant cell tumour	IL-34 promotes RANKL-induced osteoclastogenesis	[20]
Mammary cancer	IL-34 enhances M-CSF1-R–dependent macrophage infiltration and promotes tumour growth	[39]
	Lung and brain metastases derived from breast cancer express M-CSF-1 and IL-34	[39]
	Expression of IL-34 is associated with a favourable prognosis in luminal and HER2, but not basal, breast cancer patients	[40]
Human ovarian cancer	High IL-34 expression correlates with worse survival in patients with ovarian cancer	[41]
Hepatocellular carcinoma	IL-34 promotes HCC cell growth and metastasis	[42]
	High IL-34 serum levels associate with poor prognosis in patients with non-viral HCC	[43]
Osteosarcoma	IL-34 enhances osteosarcoma growth and metastasization	[44]
Cholangiocarcinoma	IL-34 promotes stemness features in cancer stem cells	[45]
Lung cancer	IL-34 and M-CSF-1 expression correlates with advanced tumour stages and poor survival in lung cancer patients	[46]
	IL-34 enhances the tumorigenic and immunosuppressive functions of TAMs and promotes the survival of chemoresistant cancer cells	[47]
Multiple myeloma	IL-34 accelerates osteoclast formation increasing the severity of bone lesions	[48]
Sporadic vestibular schwannoma	IL-34 is expressed in sporadic vestibular schwannoma	[49]
Adult T-cell leukemia/lymphoma	IL-34 co-expression with M-CSF-1 may be related to the aggressiveness of this cancer type	[50]
Refractory melanoma	Enhanced expression of IL-34 in refractory melanoma tissues	[51]

*RANKL* receptor activator of nuclear factor kappa-Β ligand, *M-CSF1-R* macrophage colony-stimulating factor-1 receptor, *HCC* hepatocellular carcinoma, *TAMs* tumour-associated macrophages, *M-CSF-1* macrophage colony-stimulating factor-1.

IL-34 is detectable at low concentrations in serum/plasma, cerebral spinal fluid, synovial fluid, and saliva [35, 55, 56] and there exists a correlation between levels of IL-34 secreted into these extracellular biofluids and disease parameters in rheumatoid arthritis, heart failure, viral infections, sepsis, periodontal disease, non-alcoholic fatty liver disease, obesity, and type 2 diabetes mellitus [35, 55–57]. Ding and colleagues showed that, in Chinese patients with rheumatoid arthritis, low serum level of IL-34 (≤194.12 pg/ml) at baseline was a good predictor of response at 3-month following anti-TNF-α treatment [58].

## Expression of IL-34 in colorectal cancer

As mentioned above, colon carcinogenesis is a tightly controlled phenomenon in which many cell types contribute to either stimulate or suppress CRC cell proliferation and death through the production of cytokines. We have recently shown that IL-34 is constitutively produced by human intestinal LPMC and its production is markedly increased in patients with IBD [26]. Moreover, high expression of IL-34 RNA and protein was found in tumour samples of patients with sporadic CRC as compared to non-tumour samples of the same CRC patients and normal controls [59]. In tumour areas, IL-34 was mostly produced by cancer cells and to lesser extent by mucosal mononuclear cells. Our observations are in line with those published by Kobayashi and co-workers, who documented high levels of IL-34 mRNA transcripts in various CRC cell lines including SW48, SW480, SW620, SW948, Caco2, CoLo205, and HT29 as compared to fetal human colon cells [60]. IL-34 mRNA was also overexpressed in primary CRC tissues taken from a cohort of 292 Japanese patients compared to the normal colorectal epithelium [60]. In the same study, the authors evaluated the impact of IL-34 expression in cancer tissues on patients’ survival. There was a strong positive correlation between high expression of IL-34 and unfavourable prognosis and poor survival. Similar findings were seen in a cohort of CRC patients registered at The Cancer Genome Atlas [60, 61]. In contrast, Wang and co-workers showed that lower expression of IL-34 gene was associated with poor survival in a cohort of 55 CRC patients [62].

Taken together these findings indicate that CRC cells produce IL-34 and suggest the possible prognostic value of the cytokine in this neoplasia.

## IL-34 as a regulator of colon cancer cell growth

**Fig. 2:**
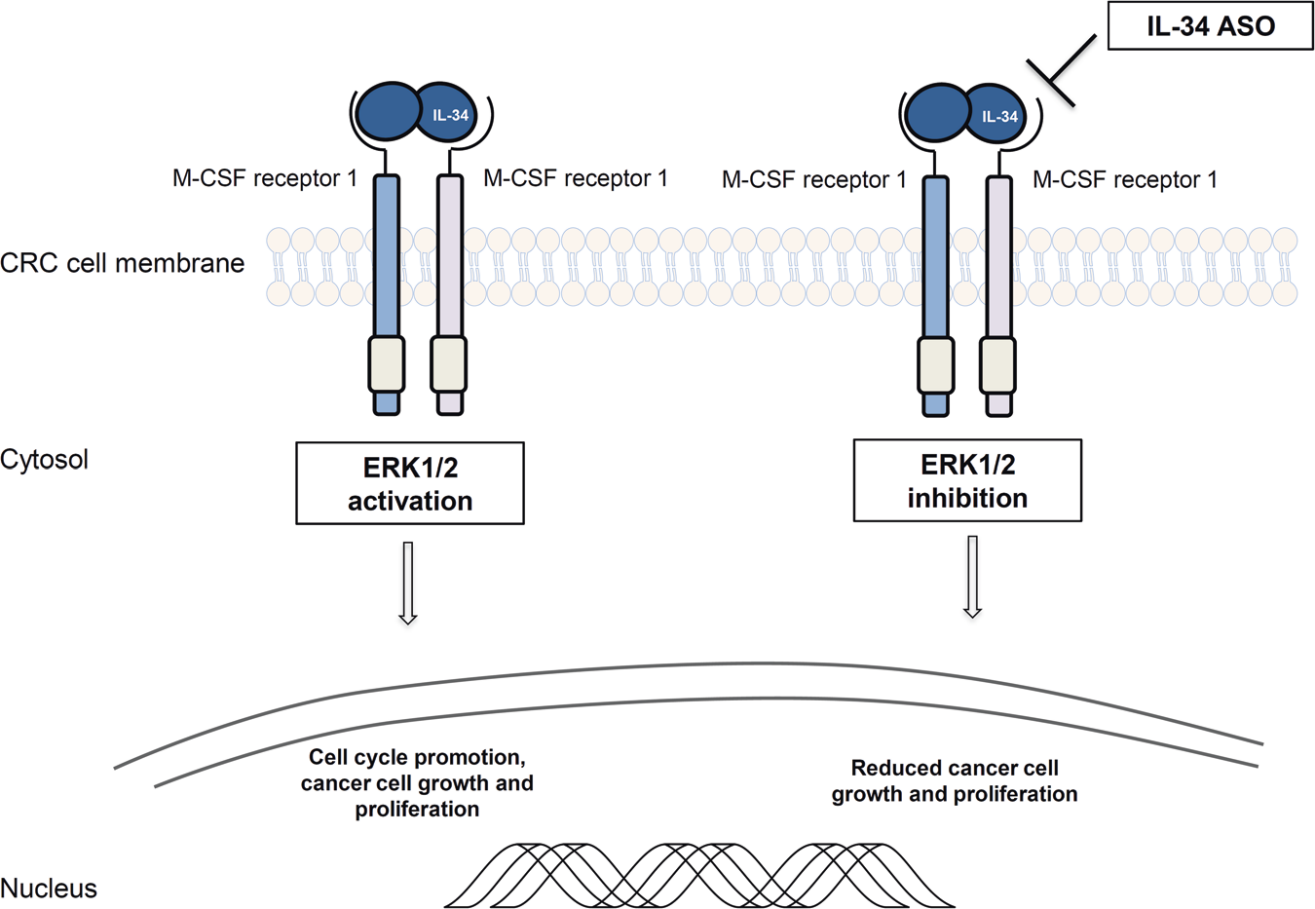
Schematic view of IL-34 as a regulator of CRC cells proliferation. IL-34 knockdown in CRC cells with an antisense oligonucleotide inhibits ERK1/2 thereby reducing cell proliferation. CRC colorectal cancer, ASO antisense oligonucleotide.

M-CSFR-1 expression was found to be more pronounced in the tumour areas as compared to the non-tumour areas of CRC patients and immunohistochemical studies showed that CRC cells were strongly positive for this receptor [59]. PTP-ζ was also constitutively expressed in the human colon with no apparent difference between tumour and non-tumour samples [59]. Altogether these data support the hypothesis that CRC cells are functionally able to respond to locally produced IL-34. Indeed, stimulation of CRC cells with recombinant IL-34 resulted in enhanced CRC cell proliferation and migration [59]. No change in cell growth was seen in normal intestinal epithelial cells following IL-34 stimulation, clearly indicating that the proliferative effect of IL-34 is restricted to the neoplastic cells. The mitogenic effect of IL-34 on CRC cells was preventable by a pharmacologic inhibitor of ERK1/2 MAP kinase pathway. Consistent with this, IL-34 knockdown in CRC cells with an antisense oligonucleotide (ASO) inhibited ERK1/2 activation, thereby resulting in reduced cell proliferation [59] (Fig. 2). In contrast, IL-34 did not affect the rate of apoptosis/necrosis of CRC cells either left untreated or treated with FAS Ligand or TNF. However, IL-34 knockdown enhanced the susceptibility of CRC cells to oxalipaltin-induced death, in line with the demonstration that IL-34, produced during chemotherapy, increases lung cancer cell survival [47].

IL-34 activated also p38 MAP kinases in CRC cells but pharmacological inhibition of this pathway did not influence the mitogenic effect of IL-34. It is, however, conceivable that IL-34-induced p38 MAP kinase activation can contribute to regulate other CRC cell functions. In this context, it is noteworthy that IL-34 activates p38 MAP kinase signal pathway in bone-marrow-derived macrophages thus resulting in enhanced expression of CD36 [63], a scavenger receptor for fatty acid uptake that modulates cell-to-extracellular matrix attachment and has pro-metastatic functions in several cancers [64].

## IL-34 and tumour-associated macrophages (TAMs)

Tumour-associated macrophages (TAMs) are one of the most abundant immune cell populations in the tumour microenvironment [65]. TAMs can be differentiated into two main subsets with distinctive phenotypes and functions, referred to as M1 (or classic) and M2 (or alternative). M1 macrophages are pro-inflammatory, while M2 macrophages are anti-inflammatory, and this distinction corresponds, respectively, to the anti-tumour and pro-tumour functions of such cell types in the tumour microenvironment [66]. Indeed, M2 stimulate tumour cell proliferation, migration, invasion, and metastasis and high numbers of these cells often correlate with a bad prognosis and therapeutic resistance [67]. TAMs originate mainly from the blood compartment and chemotactic factors produced by tumour cells or by normal cells present in the cancer microenvironment enhance recruitment of monocytic precursors at the tumour site. Then, differentiation and activation of TAMs is favoured by further factors released by cancer cells, stromal cells and/or immune cells [67]. IL-34 promotes differentiation of monocytes into IL-10-expressing, immunoregulatory macrophages, which exhibit similarities to TAMs seen in ovarian cancer [68, 69]. Moreover, macrophages stimulated with IL-34 promote differentiation of CCR4 + CCR6 + CD161 + Th17 cells, a phenomenon occurring in many cancers [67]. Overall, these findings raise the possibility that IL-34 can contribute to the differentiation and activation of TAMs in CRC. This hypothesis is also supported by the demonstration that, in CRC, IL-34 expression correlates with the content of CD163, a marker of TAMs [60]. By flow-cytometry analysis of tumour-infiltrating cells (TICs) and LPMC isolated from normal adjacent mucosa of CRC patients, we have recently shown that CD68/HLA-DRII-expressing TICs and LPMC expressed M-CSF-1-R [70]. Both these cell types produced IL-34 even though IL-34 expression was more pronounced in TICs as compared to normal LPMC. IL-34 was produced by CD68/HLA-DRII-positive cells either expressing or not M-CSF-1-R, suggesting that IL-34 can regulate TAMs functions by acting in a paracrine and/or autocrine manner. Indeed, stimulation of both TICs and LPMCs with IL-34 enhanced the expression of CD163 and CD206, two markers of type-2-polarized macrophages [71]. Moreover, stimulation of both TICs and LPMCs with IL-34 enhanced IL-6 synthesis [70], a cytokine that activates proliferative and survival signals in CRC cells. Consistently, knockdown of IL-34 in TICs with a specific ASO decreased IL-6 production and the number of IL-6-producing TAMs [70]. These findings are in agreement with the demonstration that expression of IL-34 is associated with increased infiltration and function of type-2-polarized TAMs in other cancer types [72].

## IL-34 and cancer-associated fibroblasts

**Fig. 3:**
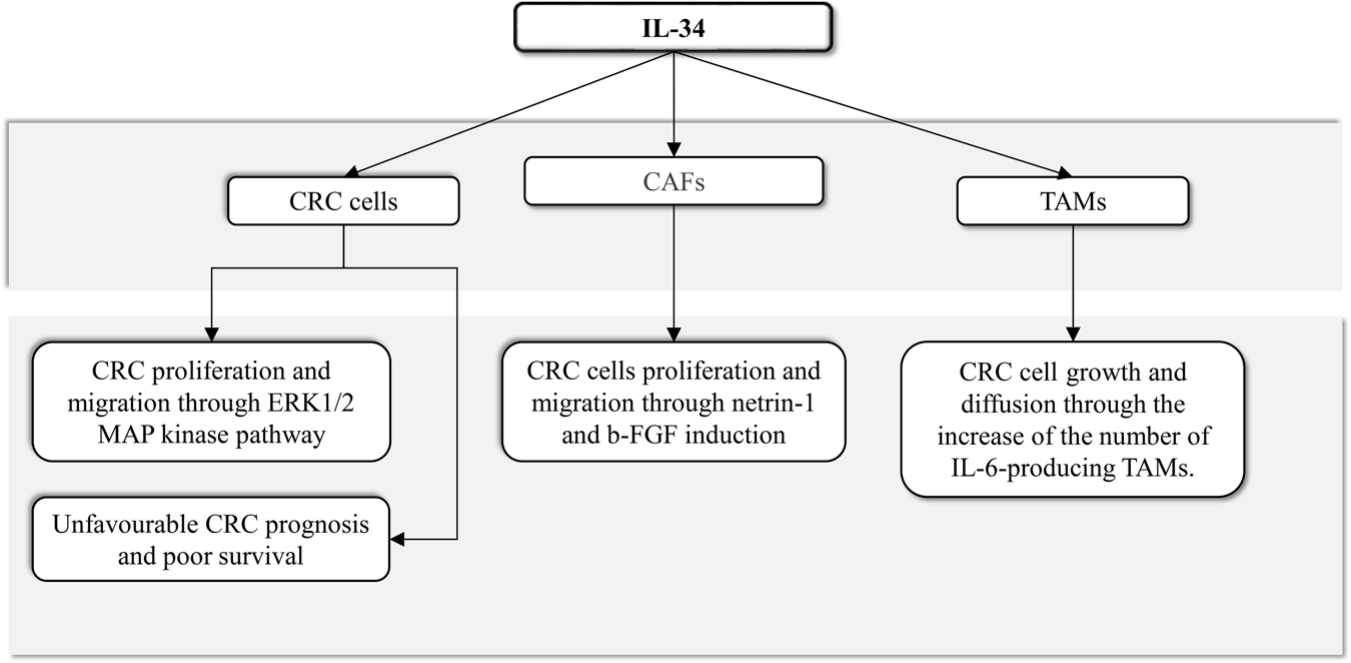
Schematic view of interleukin-34 effects on specific cell types in CRC microenvironment. CRC colorectal cancer, TAMs tumour-associated macrophages, CAFs cancer-associated fibroblasts, b-FGF basic fibroblast growth factor.

The stromal compartment of CRC contains numerous activated fibroblasts, termed cancer-associated fibroblasts (CAFs), which promote CRC growth and progression, resistance to chemotherapy, and relapse of cancer through the synthesis of various molecules targeting the neoplastic cells [73, 74]. By real-time PCR, immunohistochemistry, and flow-cytometry we showed that IL-34 RNA transcripts and protein were significantly increased in CAFs compared to the fibroblasts isolated from the normal, adjacent colonic mucosa of the same patients with sporadic CRC [75]. IL-34 was also abundantly expressed in CAFs isolated from ulcerative colitis-associated CRC as compared to normal fibroblasts [75]. Moreover, CAFs and normal fibroblasts expressed both M-CSFR-1 and PTP-ζ [75]. Our data indicate also that, in the human gut, IL-34 promotes differentiation of CAFs with tumorigenic properties. Indeed, stimulation of normal colonic fibroblasts with IL-34 enhanced the expression of typical markers of CAFs, such α-SMA, Vimentin, and fibroblast activation protein (FAP) and induced cell proliferation, while inhibition of IL-34 expression in CAFs with a specific ASO decreased the expression of CAFs markers and proliferation [75]. CRC cells cultured in the presence of IL-34 AS-treated CAFs supernatants exhibited a significant reduction in proliferation as compared to CRC cells cultured in the presence of supernatants of CAFs treated with control AS. Moreover, a scratch test revealed that the supernatants of IL-34 AS-treated CAFs had reduced ability to stimulate CRC cell migration as compared to the supernatants of untreated CAFs [75]. These findings suggest that IL-34 stimulates CAFs to synthesize factors promoting CRC cell proliferation and migration (Fig. 3). Indeed, IL-34 regulates positively expression of netrin-1 and basic fibroblast growth factor (b-FGF) [75]. Netrin-1 is a multifunctional secreted glycoprotein involved in the control of several biological processes, such as angiogenesis, neuronal navigation, cell survival, and migration, and accumulating evidence suggests a role for this protein in many pathologies, including cardiovascular diseases, diabetes, and cancer [76–78]. Netrin-1 is highly expressed by CAFs in CRC tissue and regulates CRC cell stemness [79, 80]. Similarly, b-FGF stimulates the acquisition of metastatic capacity by CAFs and regulates positively the growth of CRC cells [81].

## IL-34 in other types of cancer

As already stated above, an elevated expression of IL-34 was seen in other cancer types. For example, a higher expression of IL-34 was found in lung cancer compared to normal lung tissues and this was correlated with a poor patients’ prognosis [46]. Moreover, in lung adenocarcinoma, IL-34 protein levels increased upon treatment with doxorubicin and cisplatin, suggesting a possible regulation of IL-34 in response to therapy [47]. Elevated levels of IL-34 were also documented in cell lines of human osteosarcoma and were associated with disease progression [20]. Many other studies documented the contribution of IL-34 in other malignancies, such as haematological tumours, brain, breast, neck, biliary, and ovarian cancers (Table 2).

## Conclusions

Studies in human CRC tissue and experimental models of cancer support the view that locally produced cytokines regulate critical steps of the colon carcinogenesis [82, 83]. The findings discussed in this article suggest that IL-34 regulates positively the function of CRC cells as well as other immune cells and non-immune cells in the CRC microenvironment, with the downstream effect of enhancing the growth and invasion of CRC cells [59, 70, 75], (Fig. 3). The possibility to use IL-34 inhibitors to block its pro-tumorigenic effects could thus open up a challenging opportunity for a new treatment option in CRC [59, 70, 75]. In this context, however, further work is needed to confirm the in vitro data generated using cells isolated from human CRC samples in pre-clinical models of CRC and ascertain whether IL-34 blockers can enhance the properties of other anti-tumoural drugs, including chemotherapeutics and biologics. It remains, also, unclear whether the marked expression of IL-34 in CRC tissue is paralleled by high circulating levels of the cytokine and whether IL-34 may serve as a prognostic biomarker in this neoplasia. Indeed, elevated levels of IL-34 have been associated with poor prognosis in primary lung cancer and such levels significantly correlate with the development of chemoresistance and progression in non-viral hepatocellular carcinoma and in basal breast cancer [40, 43, 46].

We also need to know much more about the factors/mechanisms underlying the high expression of IL-34 in CRC and to ascertain if there exists a cell-specific regulation of IL-34. Since M-CSF-1-R is expressed by additional cell types other than cancer cells, macrophages, and fibroblasts, it is likely that IL-34 can regulate the function of other immune and non-immune cells in the CRC microenvironment [84]. The fact that PTP-ζ is expressed in CRC tissue suggests that this receptor can mediate additional functions of the cytokine. Another possibility is that following IL-34 stimulation, signals driven by both PTP-ζ and M-CSF1-R are necessary to influence CRC cell behaviour. Support to this hypothesis comes from the observation that no change in CRC cell proliferation and survival was seen in cultures stimulated with M-CSF-1, the other ligand of M-CSF1R.

Overall, the data here described underline the role of IL-34 in positively regulate the function of CRC cells as well as other cell types (i.e. TAMs and CAFs), which ultimately sustain CRC cell behaviour. It is relevant to point out that the pro-tumorigenic role of IL-34 could also rely on additional functions of the cytokine. For instance, IL-34 induces immune cells to produce TNF-α [26], a cytokine exerting proliferative effects on CRC cells [85]. IL-34 has also been involved in the suppressive function of regulatory T cells, the activity of which associates with the progression of CRC cancer cells [86]. Finally, IL-34 stimulates macrophages to switch non-Th17 committed memory CD4(+) T cells into Th17 cells [87], which are known to enhance CRC cell growth and migration [88].

## Supplementary information

Declaration of Contribution to article
