# Supplementary material for: Interleukin-34 promotes tumorigenic signals for colon cancer cells
Source: Cell Death Discov. 2021 Sep 17;7:245. doi: 10.1038/s41420-021-00636-4 (PMC8448832; doi:10.1038/s41420-021-00636-4)
Supplement: Supplementary file 1 — Declaration of Contribution to article [file 41420_2021_636_MOESM1_ESM.pdf]

DECLARATION OF CONTRIBUTIONS TO ARTICLE

ADMC

Manuscript Number: -21-1668R

Journal Name: *Cell Death Discovery* (the 'Journal')

Proposed Title of the Contribution:

INTERLEUKIN-34 PROMOTES TUMORIGENIC SIGNALS FOR COLON CANCER CELLS (the 'Contribution)

Author(s):

ELEONORA FRANZÈ, IRENE MARAFINI, EDOARDO TRONCONE, SILVIA SALVATORI, GIOVANNI MONTELEONE (the 'Authors')

For all *CDDis*covery articles, each person named as an author in the published version must be able to show he or she has contributed substantially to the article.

Authorship credit should be based on 1) substantial contributions to conception and design, acquisition of data, or analysis and interpretation of data; 2) drafting the article or revising it critically for important intellectual content; and 3) final approval of the version to be published. Authors should meet conditions 1, 2 and 3.

Any person who cannot be shown to have made a substantial contribution to the article cannot be listed as an author in the final version. The name of any person who is deemed to have made a minor contribution can, however, appear in the Acknowledgments section of the article.

Please complete the table below to indicate the contributions of all named authors to the manuscript.

Author Full Name:	Specification of Contribution to the Manuscript:
ELEONORA FRANZÈ	ACQUISITION OF DATA, REVISION OF THE ARTICLE, FINAL APPROVAL
IRENE MARAFINI	ACQUISITON OF DATA, DRAFT OF THE ARTICLE, FINAL APPROVAL
EDOARDO TRONCONE	ACQUISITION OF DATA, REVISION OF THE ARTICLE, FINAL APPROVAL
SILVIA SALVATORI	ACQUISITION OF DATA, REVISION OF THE ARTICLE, FINAL APPROVAL
GIOVANNI MONTELEONE	ACQUISITION OF DATA, CONCEPTION AND DESIGN, DRAFT, FINAL APPROVAL

Please complete the table below to indicate the contributions of all named authors to the figures.

Figure 1:

E.F. : CONCEPTION, FINAL APPROVAL
I.M. : CONCEPTION, FINAL APPROVAL
E.T. : CONCEPTION, FINAL APPROVAL
S.S. : CONCEPTION, FINAL APPROVAL
G.M. : CONCEPTION AND DESIGN, FINAL APPROVAL

Figure 2:

E.F: CONCEPTION, FINAL APPROVAL
I.M.: CONCEPTION AND DESIGN, FINAL APPROVAL
E.T.: CONCEPTION, FINAL APPROVAL
S.S.: CONCEPTION, FINAL APPROVAL
G.M.: CONCEPTION, FINAL APPROVAL

Figure 3:

E.F: CONCEPTION AND DESIGN, FINAL APPROVAL
I.M.: CONCEPTION, FINAL APPROVAL
E.T.: CONCEPTION, FINAL APPROVAL
S.S.: CONCEPTION, FINAL APPROVAL
G.M.: CONCEPTION, FINAL APPROVAL

Figure 4:

Figure 5:

Figure 6:

Signed for and on behalf of the Author(s):	Print Name:	Date:
[signature]	GIOVANNI MONTELEONE	26/05/2021
